# Enterovirus A71 Infection, Thailand, 2017

**DOI:** 10.3201/eid2407.171923

**Published:** 2018-07

**Authors:** Jiratchaya Puenpa, Chompoonut Auphimai, Sumeth Korkong, Sompong Vongpunsawad, Yong Poovorawan

**Affiliations:** Chulalongkorn University, Bangkok, Thailand

**Keywords:** enterovirus, enterovirus A71, coxsackievirus, viruses, infection, hand, foot and mouth disease, myocarditis, meningitis/encephalitis, outbreak, Thailand

## Abstract

An outbreak of hand, foot and mouth disease among children in Thailand peaked in August 2017. Enterovirus A71 subgenogroup B5 caused most (33.8%, 163/482) cases. Severe disease (myocarditis and encephalitis) was observed in 1 patient. Coxsackievirus A6 was detected in 6.0% (29/482) of patients, and coxsackievirus A16 was detected in 2.7% (13/482) of patients.

Hand, foot and mouth disease (HFMD) and herpangina, caused primarily by enterovirus A, commonly affect children and result in painful blisters in the buccal cavity and on the soles of the hands and feet. In rare situations, enterovirus infection can lead to severe neurologic complications, notably aseptic meningitis, encephalitis, acute flaccid paralysis, and death in young children (*1*).

A nationwide outbreak of HFMD caused by coxsackievirus A6 (CV-A6) affected many children in Thailand in 2012 (*2*). Since that time, ≈50,000 cases of HFMD have been reported annually to the Thailand Ministry of Public Health (*3*); CV-A6 and CV-A16 are the main causative agents. Most reports were based on clinical symptoms; laboratory-based confirmations are rare. Consequently, the incidence increased awareness and the need for diagnostic-based epidemiologic surveillance. As of September 25, 2017, a total of 59,071 cases of HFMD have been reported (*4*). We determined the prevalence and viral etiology of enterovirus infections among patients with clinical HFMD and herpangina in Thailand during 2017.

The study was approved by the Chulalongkorn University Faculty of Medicine Institutional Review Board (institutional review board no. 002/60). We tested clinical specimens from 482 children requiring hospital care for HFMD (n = 435) and herpangina (n = 47) that were submitted to King Chulalongkorn Memorial Hospital (Bangkok, Thailand) during January 1–October 30, 2017, from 12 provinces with reported HFMD outbreaks (Technical Appendix Figure 1). The study population was 273 boys and 209 girls (sex ratio M:F 1.3:1; age range 4 days to 45 years; mean ± SD age 2.9 ± 4.0 years; median age 2.0 years).

We subjected specimens to 2 real-time reverse transcription PCRs (RT-PCRs). The first RT-PCR identifies enterovirus A71 (EV-A71), CV-A6, and CV-A16 (*5*). We then subjected virus-positive samples to full-length virus capsid protein 1 (VP1) gene amplification by using a conventional RT-PCR and nucleotide sequencing to identify EV-A71 subgenogroups (*6*). The second RT-PCR is a pan-enterovirus real-time RT-PCR, which also detects the glyceraldehyde-3-phosphate dehydrogenase gene (internal control). We subjected samples to a conventional RT-PCR that used CODEHOP degenerate primers to identify enterovirus serotypes other than EV-A71, CV-A6, and CV-A16 (*7*). We also performed molecular typing by using phylogenetic analysis and nucleotide sequence comparisons of strains. Sequences were deposited in GenBank (accession nos. MG843892–844136).

Enterovirus infections were detected throughout the year; increased frequency was observed in the rainy season (June–September) (Technical Appendix Figure 1). Enteroviruses were identified in 67.4% (325/482) of samples. EV-A71 was most frequent (33.8%, 163/482), followed by CV-A6 (6.0%, 29/482), and CV-A16 (2.7%, 13/482). Analysis of partial VP1 sequences showed that almost all (99.4%, 162/163) of EV-A71 was subgenogroup B5: only 2 samples were subgenogroup C4 (Technical Appendix Figure 1A). Full-length VP1 analysis also confirmed our EV-A71 subgenogroup assignment (Technical Appendix Figure 2).

Comparison of full-length VP1 nucleotide sequences of subgenogroup B5 viruses between strains isolated in Thailand during this study and those previously isolated in Thailand showed 93.3%–100.0% identity (Technical Appendix Table). Strains isolated in this study also had similar sequence identity with strains previously isolated (92.7%–98.6%). CV-A4 accounted for 6.6% (32/482) of isolates, followed by CV-A2 (1.5%, 7/482) and CV-A8 (1.0 %, 5/482). Minor genotypes (CV-A5, CV-A10, and echovirus 9) accounted for 0.4% (2/482). One sample each was positive for CV-A9, CV-B2, echovirus 4, and echovirus 5. The remaining 13.5% (65/482) of isolates positive for pan-enterovirus were not typeable.

A severely ill 9-month-old boy in Nakhon Sawan Province was given a diagnosis of HFMD and required prolonged hospitalization because of sequelae caused by severe brain damage from encephalitis and myocarditis. Laboratory tests on rectal swab samples identified EV-A71 subgenogroup B5. An 18-month-old girl from Uttaradit Province who suddenly died during development of HFMD was positive for CV-A9.

Enteroviruses associated with HFMD in Thailand during 2012–2016 are usually CV-A6 or CV-A16 (*5*). EV-A71 has been associated with major outbreaks in China and Vietnam (*8*), and infections have been linked to more severe symptoms and neurologic complications in patients requiring hospitalization. In the HFMD outbreak in Thailand during 2012, deaths were primarily associated with EV-A71 subgenogroup B5 (*9*). The relatively high frequency of HFMD attributed to EV-A71 in this study was unexpected because our previous studies showed that <10% of enterovirus-positive samples were EV-A71 (*2*,*9*).

Our study had limitations. Because samples were obtained only from provinces with reported HFMD outbreaks, we might have missed HFMD-associated enterovirus in other regions. Limited availability of demographic and clinical data also prevented more in-depth analysis of disease severity and clinical associations. Additional deaths caused by EV-A71 infections might have been missed. Thus, without accurate and complete reporting of severe complications associated with EV-A71 infections, clinical implications from such infections could not be determined.

Effective prevention of widespread HFMD is needed. The approved EV-A71 vaccine containing subgenogroup C4 and ongoing development of B2- and B4-based vaccines can elicit cross-neutralizing antibodies against circulating EV-A71 subgenogroups, which might prove to be effective in preventing subgenogroup B5 infection, which is predominant in Thailand (*10*). However, molecular epidemiologic surveillance of HFMD is needed to monitor enterovirus transmission.

Technical Appendix Additional information on enterovirus A71 infection, Thailand, 2017.
