# Supplementary material for: Enterovirus A71 Infection, Thailand, 2017
Source: Emerg Infect Dis. 2018 Jul;24(7):1386–7. doi: 10.3201/eid2407.171923 (PMC6038748; doi:10.3201/eid2407.171923)
Supplement: Technical Appendix — Additional information on enterovirus A71 infection, Thailand, 2017. [file 17-1923-Techapp-s1.pdf]

Article DOI: https://doi.org/10.3201/eid2407.171923

Enterovirus A71 Infection, Thailand, 2017

Technical Appendix

Technical Appendix Table. Comparison of nucleotide sequence identity of full-length VP1 gene of enterovirus A71 subgenogroup B5 with other virus strains*

Sequence	CU2637	CU2412	CU2202	TH2009	TH2011	TH2013	SG2008	CN2009	MY2010	KH2012	TW2012	FR2013
CU2412	96.5											
CU2202	96.4	99.2										
TH2009	93.7	94.6	94.5									
TH2011	93.3	94.0	94.1	97.6								
TH2013	96.5	97.5	97.6	95.0	94.0							
SG2008	94.0	94.9	94.8	99.2	97.9	95.1						
CN2009	94.9	95.9	96.0	95.0	94.0	96.8	95.3					
MY2010	92.9	93.6	93.4	96.5	95.5	93.8	96.8	94.2				
KH2012	92.7	93.8	93.7	97.4	98.6	94.0	97.7	93.8	95.2			
TW2012	94.2	95.2	95.3	94.6	94.0	96.1	94.9	97.3	94.5	94.0		
FR2013	96.8	98.5	98.6	94.9	94.3	98.3	95.2	96.5	93.7	94.1	95.8	
VN2013	96.5	97.7	97.8	94.8	94.0	98.4	95.1	96.6	93.8	93.8	95.9	98.5

*Values are percentage identities. Blank spaces indicate 100% identity. Strains used in phylogenetic trees and their GenBank accession numbers are TH/2009 (JF738001), TH/2011 (JQ900615), TH/2013 (KM675914), SG/2008 (FJ461783), CN/2009 (JN964686), MY/2010 (KC894872), KH/2012 (KP308430), TW/2012 (KF154339), FR/2013 (LK985324), and VN/2013 (KU888136). CN, China; FR, France; KH, Cambodia; MY, Malaysia; SG, Singapore; TH, Thailand; TW, Taiwan; [illegible] Vietnam; VP1, virus capsid protein 1. The VP1 sequences of enterovirus A71 obtained in this study are CU2637, CU2412, and CU2202.

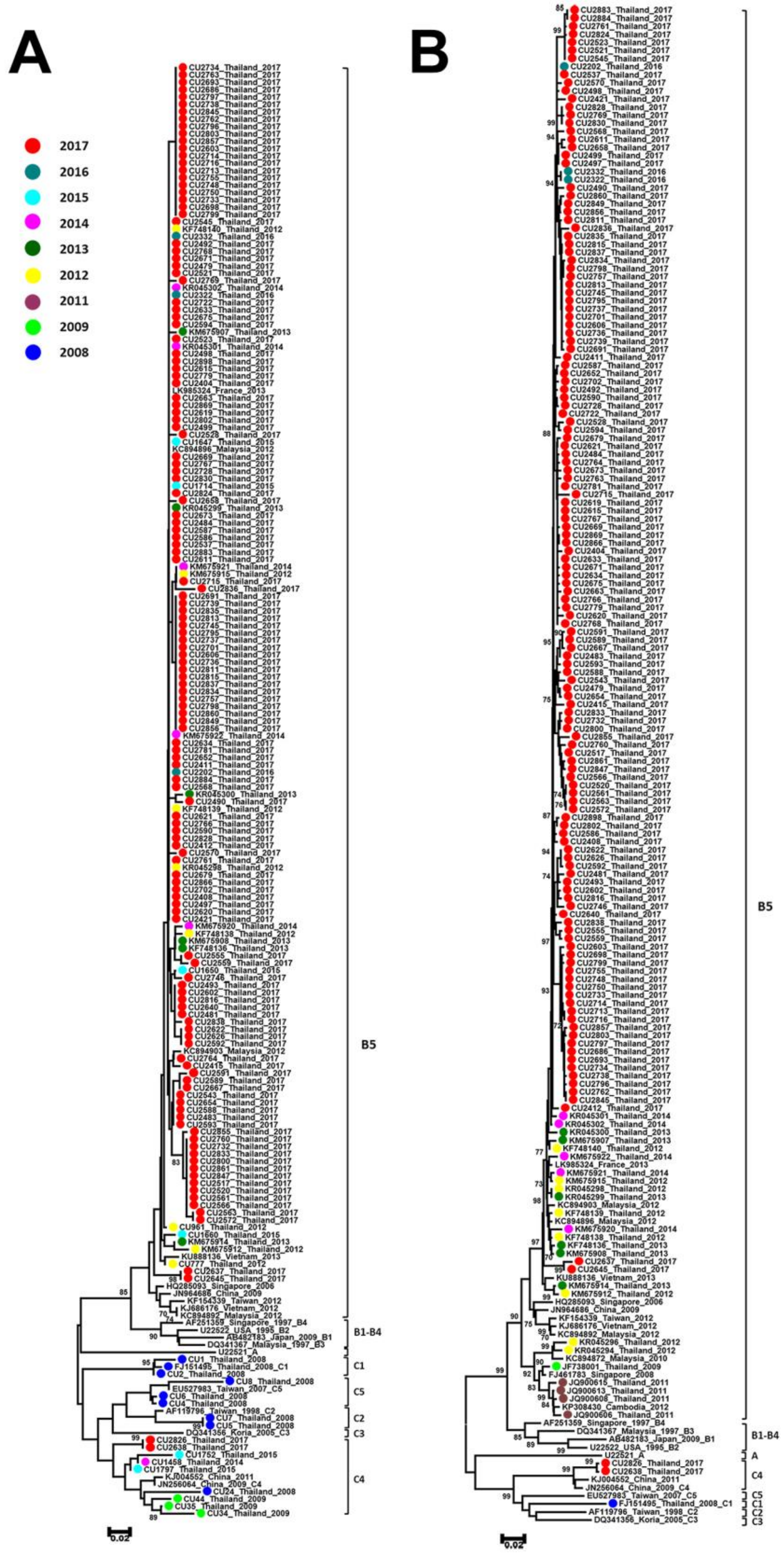

Technical Appendix Figure 1. Distribution of enterovirus-positive samples from case-patients with hand, foot, and mouth disease and herpangina, Thailand, January–October 2017. CV, coxsackievirus; EV, enterovirus.

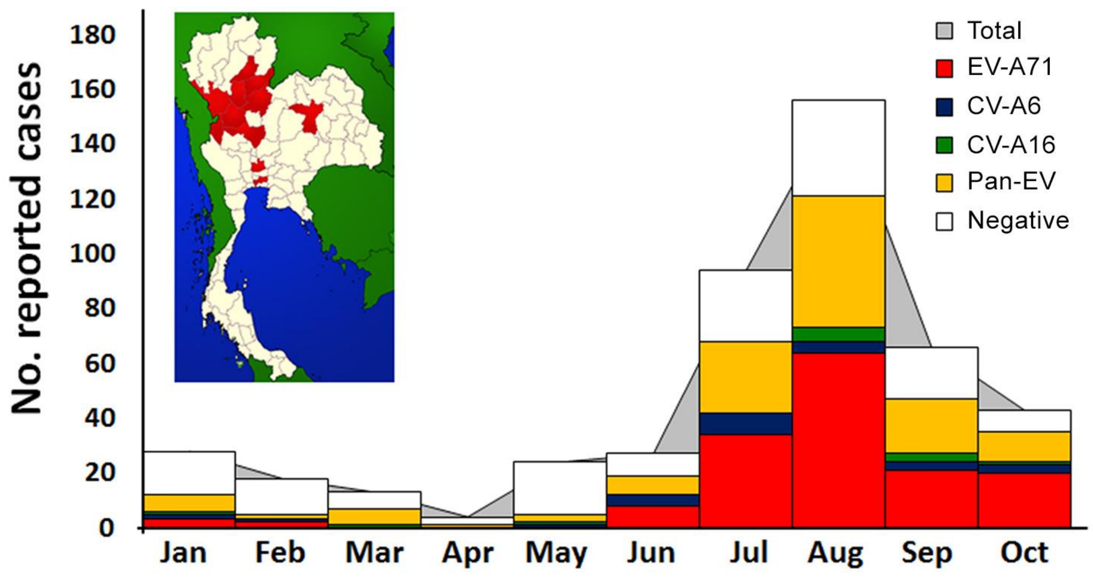

Technical Appendix Figure 2. Phylogenetic analysis of A) partial and B) complete virus capsid protein 1 sequences of enterovirus A71. Virus strains were isolated from patients with hand, foot and mouth disease during 2008–2017, including those from this study, and compared with references strains from GenBank. The tree was constructed by using the neighbor-Joining method implemented in MEGA6 (https://www.megasoftware.net/). Virus strains characterized in this study are indicated by red circles. Only bootstrap resampling values ≥70% are shown. Scale bars indicate nucleotide substitutions per site.
